# Microbial Musings – November 2020

**DOI:** 10.1099/mic.0.001005

**Published:** 2020-11-30

**Authors:** Gavin H. Thomas

**Affiliations:** ^1^​ Department of Biology, University of York, PO Box 373, York, UK

This month has been a very exciting one for the journal as we have been proactively redeveloping our topic areas and appointing new Senior Editors and Editors to take the journal forward into an exciting 2021. I’ll bring you this exciting news and our new faces in the December *Microbial Musings*. First off this month, I’d like to congratulate Elizabeth Bik (@MicrobiomDigest), who has been awarded the 2021 Peter Wildy Prize of the Microbiology Society for her outstanding work as a communicator of microbiome research and, more recently – and highly relevant to the journal and scientific publishing more generally – her image sleuthing work. I am in awe of her ability to spot image duplications that I would never have spotted, but once she has pointed out are so obvious that one cannot understand why one did not see them in the first place! Her landmark publication from a survey of over 20 000 papers in the biomedical field found inappropriate image manipulation in around 4 % of these, which is 4 % more than should be acceptable to our community [1]. We have worked with Dr Bik and PubPeer (https://pubpeer.com/) to address a the few examples of these from papers published in *Microbiology*, following the guidance of the Committee of Publication Ethics (COPE), which the Microbiology Society is a member of, and I hope that other microbiology journals will put in similar efforts to correct the scientific record. I look forward to her prize lecture in 2021 with relish.

We start the monthly review with a Microbe Profile on the opportunistic fungal pathogen *Cryptococcus neoformans* [2], written by Xiaorong Lin and colleagues at the University of Georgia, USA, with Joseph Heitman (@DukeHeitman) from Duke University, USA. They introduce this microbe as one that is present in various environmental reservoirs and has really emerged as a killer in humans since the increase in individuals living with compromised immune systems as a result of other diseases, such as HIV/AIDS and leukaemia. The fungus is well equipped to infect these patients and employs a range of mechanisms, some similar to bacterial pathogens such as a complex sugar capsule that has roles in immune evasion [3], and others more unique to this fungus such as the use of host dopamine to synthesize melatin that is added as another defensive molecule into the cell wall [4]. As they cannot move between humans, infections come from the environment and the fungus can often stay dormant and reactivate at a later stage. How this is controlled and how this might be prevented are two of the open questions for future researchers to address to try and control this dangerous pathogen.

The second article to highlight is a fascinating review on expansin-like proteins made by microbes that interact with plants from Claudia Martinez-Anaya and colleagues at the Universidad Nacional Autónoma de México, Mexico. These are similar to plant expansins that are able to loosen the plant cell wall through a poorly defined non-enzymatic slipping mechanism [5]. Structurally they look like carbohydrate active enzymes, containing a pair of glycoside hydrolase (GH) and carbohydrate-binding module (CBM) domains, where the GH domain appears to have lost enzymatic activity, leaving the binding function alone. For some plant pathogens such as *Ralstonia solanacerum* they are important for xylem infection [6], presumably by helping to gain entry across the plant cell wall. The review discusses the diversity and function of these proteins, along with related swollenins that are only seen in ascomycete fungi. Staying with plant and soil microbiology, there is an interesting short communication from the group of Suvit Loprasert from the Chulabhorn Research Institute, Bangkok, Thailand, who have made an improved whole-cell biosensor for the pesticide chlorpyrifos, which uses the *Sinorhizobium meliloti* transcriptional activator ChpR and the *chpA* promoter moved onto the *Escherichia coli* chromosome, producing a very high-affinity sensor [7]. Interestingly, when they add an efflux pump inhibitor they see even higher sensitivity, suggesting that the pesticide is being actively removed by *E. coli *.

The fundamental process of homologous recombination underpins many of our genetic tools for microbial manipulation and the routes by which microbes introduce DNA onto their chromosomes. The widespread protein RecA is important for efficient recombination and this works with other components of the recombination machinery to prepare and exchange DNA between the donor and acceptor. In bacteria the other major components are the RecBCD proteins in *E. coli* and the AddAB proteins in *Bacillus subtilis* and *Staphylococcus aureus* (known as RexAB), which are distinct but related proteins that serve similar functions. In this paper from the lab of Robert Blumenthal at the University of Toledo, USA, the authors make the most comprehensive analysis of the distribution of RecBCD and AddAB proteins across bacterial phyla [8]. In doing so they find broadly that many phyla contain genomes with one or the other of these two types, but in addition find a number of exceptions that suggest both horizontal transfer of these genes and potential hybrids between Rec and Add systems. The important functions of AddAB family proteins were recently highlighted by Kam Pou Ha (@kampouha) and colleagues from Andrew Edward’s (@bugsinblood) group at Imperial College London, UK. The group looked at features of the pathogen *S. aureus* that enable it to survive the oxidative burst produced when they are engulfed by neutrophils in blood. They identified RexAB as being required for repair of DNA damaged by reactive oxygen species (ROS) produced by this host response [9]. In this issue Nisha Rangananthan (@drNJ_RJ) and colleagues from Edward’s group have studied the role of the general stress response in the same process in *S. aureus *, which are genes under the control of sigma factor B (σB) [10]. They demonstrate a role of σB during survival in blood and provide evidence that this is mediated through loss of mechanisms to resist ROS. In the same paper they also show that the σB regulon contains genes involved in gentamicin and ciprofloxacin resistance as stationary phase-grown cells lacking σB are significantly more sensitive to these antibiotics than the wild-type strain.

The use of microbes in industrial biotechnology is often hampered by the toxicity of the chemical product they have been engineered to produce, and in our next paper, from the group of Nick Wierckx at the Forschungszentrum Jülich, Germany, the authors investigate the evolutionary potential for bacteria to evolve tolerance to a chemical, anthranilate, that has potential as a precursor for sustainable plastic manufacturing [11]. As an aromatic compound, like some other related plastic monomers such as styrene, it is highly toxic to the producing cell. In their work they study both the Gram-positive biotechnology workhorse *Corynebacterium glutamicum* and the robust Gram-negative bacterium *Pseudomonas putida* as potential hosts. After first establishing the levels that are toxic, they use a sequential batch fermentation strategy with *P. putida* for over 150 days to evolve stable strains with threefold higher tolerance than the parental strain, although biomass yields are low compared to growth in the absence of anthranilate. Using a similar protocol for *C. glutamicum* they do not see evolution of tolerance to higher concentrations of anthranilate, as the strain can already grow with 25 g l^−1^ of anthranilate, but much faster growth, suggesting that they are seeing the evolved enhancement of an existing non-energy-dependent mechanism, probably due to the complex cell envelope structure of this actinobacterium. This is in contrast to what is seen in *P. putida *, which is likely an energy-dependent mechanism, such as efflux, which can confer high tolerance but impacts on growth yield. Of course there is one caveat here in that the toxic effects of the chemical may well be different when it is being produced intracellularly than when applied on the outside, a concept that we are actually currently investigating in Project DETOX (http://projectdetox.co.uk/).

Our last two papers are within the topic of antimicrobial resistance (AMR) and we start with a paper from Manu Singh (@manusngh93), Ellen Sykes (@ESykes7) and colleagues from the group of Ayush Kumar (@ayushkumarLab) at the University of Manitoba, Canada. Unlike the paper from his group in the June issue on the transcription factor AvnR from *Acinetobacter baumannii *, which was also our Editor’s Choice [12], this new paper is about antibiotic efflux in *Pseudomonas aeruginosa* [13]. It is well known that *P. aeruginosa* has a high basal level of resistance to multiple antibiotics and efflux is a major component of this phenotype. Like in *E. coli *, the resistance–nodulation–division (RND) family, exemplified by AcrAB, are the predominant players conferring resistance and in *P. aeruginosa* there are multiple RND pumps with known roles in efflux of clinically important molecules. The MexXY pump is one that is often derepressed in isolates from cystic fibrosis patients and is involved in aminoglycoside resistance [14]. The RND pumps are tripartite as they link to an outer-membrane factor (OMF), for example TolC in *E. coli *, which allows them to form a trans-envelope structure, leading to pumping out of the chemical across the outer membrane [15]. *P. aeruginosa* also has multiple OMFs and there is evidence that different RND pumps can crosstalk with different OMFs. In this paper the authors use a *P. aeruginosa* strain, PAO750, where multiple RND pumps have been removed, to study the effect of coupling MexXY to different OMFs. MexXY is known to operate with OprM and OprA and in their system they demonstrate that both of these OMFs can couple to MexXY to export ethidium bromide and aminoglycosides. However, interestingly, they found that for the β-lactams carbenicillin and sulbenicillin only the MexXY–OprA complex was functional *in vivo*. This surprising result suggests that the outer-membrane protein can contribute to the substrate selectivity of the whole pump and that MexXY couples to different OMFs, depending on what substrates it needs to efflux. The molecular basis of this OMF-dependent substrate switching is now the focus of their future research.

The second AMR paper is from the group of Adam Roberts (@GCAGATGCAATG) from the Liverpool School of Tropical Medicine, UK. He is well known to the Microbiology Society as he won the Outreach Prize in 2017 for his ‘Swab and Send’ project with the general public, seeking to collect new bacterial strains that might yield new antimicrobials [16]. In the work in this issue led by Alastair Hubbard (@dralhubb) the group isolate a new mutant that results in the small-colony variant (SCV) phenotype of *E. coli* in response to aminoglycoside antibiotics. These natural products are actively taken up across the inner membrane, so cells with reduced membrane potential appear to be more tolerant to them through reduced uptake, a phenotype seen with both SCVs and persister cells [17]. In fact, the addition of an energy source to persisters potentiates them again to aminoglycosides [18], a approach that has been shown more recently to also work against *P. aeruginosa* [19]. Here the group isolate an SCV from a clinical isolate of *E. coli* from Malawi by selecting for isolates that are more tolerant to the aminoglycoside gentamicin [20]. By genome sequencing the resistant isolate, they identify a single-nucleotide polymorphism (SNP) in the *hemA* gene, which results in the Phe to Leu alternation in the resulting protein that likely reduces function. Reasoning that the lack of haem, and therefore reduced potential for respiration, reduced membrane potential and reduced active uptake of gentamicin, was the cause of the phenotype, they added haem to the media and were able to restore normal growth. Further, in the absence of selection, the SNP readily reverts back to the wild-type sequence, suggesting a strong fitness cost in the absence of selection. While an SCV isolate with *hemA* mutations has not been observed previously, mutations in *hemB*, from the same metabolic pathway, have been seen before in a a clinical *E. coli* SCV isolate [21] . Also, from a much larger study isolating strains resistant to aminoglycosides, many other genes relating to oxidative phosphorylation have been identified in non-SCV lines of *E. coli *, including *hemA* itself [22].

We close by flagging the Editor’s Choice paper for this month, selected by Reviews Editor Andrew Preston, which is a paper from the lab of Jay Zhu at the University of Pennsylvania, USA, on siderophore piracy in *Vibrio cholerae *, a clever strategy used by multiple bacteria to exploit the efforts of other bacteria in their niche that synthesize and secrete iron-sequestering siderophores, which they then take up and use as a source of iron, which is often limiting for growth [23]. Read more about the paper on the Microbiology Society blog and I’ll be back in December with news of our exciting changes for 2021.

## References

[1] Bik EM, Casadevall A, Fang FC (2016). The prevalence of inappropriate image duplication in biomedical research publications. mBio.

[2] Bahn Y-S, Sun S, Heitman J, Lin X (2020). Microbe profile: *Cryptococcus neoformans* species complex. Microbiology.

[3] O'Meara TR, Alspaugh JA, Andrew Alspaugh J (2012). The Cryptococcus neoformans capsule: a sword and a shield. Clin Microbiol Rev.

[4] Eisenman HC, Mues M, Weber SE, Frases S, Chaskes S (2007). Cryptococcus neoformans laccase catalyses melanin synthesis from both D- and L-dopa. Microbiology.

[5] Narváez-Barragán DA, Tovar-Herrera OE, Segovia L, Serrano M, Martinez-Anaya C (2020). Expansin-related proteins: biology, microbe-plant interactions and associated plant-defense responses. Microbiology.

[6] Tancos MA, Lowe-Power TM, Peritore-Galve FC, Tran TM, Allen C (2018). Plant-like bacterial expansins play contrasting roles in two tomato vascular pathogens. Mol Plant Pathol.

[7] Chouichit P, Whangsuk W, Sallabhan R, Mongkolsuk S, Loprasert S (020). A highly sensitive biosensor with a single-copy evolved sensing cassette for chlorpyrifos pesticide detection. *Microbiology* 2.

[8] Gurung D, Blumenthal RM (2020). Distribution of RecBCD and AddAB recombination-associated genes among bacteria in 33 phyla. Microbiology.

[9] Ha KP, Clarke RS, Kim G-L, Brittan JL, Rowley JE (2020). Staphylococcal DNA repair is required for infection. mBio.

[10] Ranganathan N, Johnson R, Edwards AM (2020). The general stress response of *Staphylococcus aureus* promotes tolerance of antibiotics and survival in whole human blood. Microbiology.

[11] Kuepper J, Otto M, Dickler J, Behnken S, Magnus J (2020). Adaptive laboratory evolution of *Pseudomonas putida* and *Corynebacterium glutamicum* to enhance anthranilate tolerance. Microbiology.

[12] De Silva PM, Patidar R, Graham CI, Brassinga AKC, Kumar A (2020). A response regulator protein with antar domain, AvnR, in *Acinetobacter baumannii* ATCC 17978 impacts its virulence and amino acid metabolism. Microbiology.

[13] Singh M, Sykes EME, Li Y, Kumar A (2020). MexXY RND pump of Pseudomonas aeruginosa PA7 effluxes bi-anionic β-lactams carbenicillin and sulbenicillin when it partners with the outer membrane factor OprA but not with OprM. Microbiology.

[14] Guénard S, Muller C, Monlezun L, Benas P, Broutin I (2014). Multiple mutations lead to MexXY-OprM-dependent aminoglycoside resistance in clinical strains of Pseudomonas aeruginosa. Antimicrob Agents Chemother.

[15] Du D, Wang-Kan X, Neuberger A, van Veen HW, Pos KM (2018). Multidrug efflux pumps: structure, function and regulation. Nat Rev Microbiol.

[16] Roberts AP (2020). Swab and send: a citizen science, antibiotic discovery project. Future Sci OA.

[17] Stokes JM, Lopatkin AJ, Lobritz MA, Collins JJ (2019). Bacterial metabolism and antibiotic efficacy. Cell Metab.

[18] Allison KR, Brynildsen MP, Collins JJ (2011). Metabolite-enabled eradication of bacterial persisters by aminoglycosides. Nature.

[19] Meylan S, Porter CBM, Yang JH, Belenky P, Gutierrez A (2017). Carbon sources tune antibiotic susceptibility in Pseudomonas aeruginosa via tricarboxylic acid cycle control. Cell Chem Biol.

[20] Hubbard ATM, Bulgasim I, Roberts AP (2020). A novel *hemA* mutation is responsible for a small-colony-variant phenotype in *Escherichia coli*. Microbiology.

[21] Roggenkamp A, Sing A, Hornef M, Brunner U, Autenrieth IB (1998). Chronic prosthetic hip infection caused by a small-colony variant of Escherichia coli. J Clin Microbiol.

[22] Lázár V, Pal Singh G, Spohn R, Nagy I, Horváth B (2013). Bacterial evolution of antibiotic hypersensitivity. Mol Syst Biol.

[23] Byun H, Jung I-J, Chen J, Larios Valencia J, Zhu J (2020). Siderophore piracy enhances *Vibrio cholerae* environmental survival and pathogenesis. Microbiology.

