# Erratum: Microbial Musings – November 2020

**DOI:** 10.1099/mic.0.001014

**Published:** 2020-12-23

**Authors:** Gavin H Thomas

**Affiliations:** ^1^​ Department of Biology, University of York, PO Box 373, York, UK

The Microbe Profile discussed in this article (paragraph two, reference two), was published in the September Issue, Volume 166 not in the current issue (November, Volume 166).

The Microbiology Society apologizes for any inconvenience or embarrassment caused.

